# Supplementary material for: Mechanically controlled multifaceted dynamic transformations in twisted organic crystal waveguides
Source: Nat Commun. 2024 May 13;15:4040. doi: 10.1038/s41467-024-47924-y (PMC11091112; doi:10.1038/s41467-024-47924-y)
Supplement: Supplementary file 1 — Supplementary Information [file 41467_2024_47924_MOESM1_ESM.pdf]

Supplementary information

Mechanically-controlled multifaceted dynamic transformations in twisted organic crystal waveguides

Mehdi Rohullah[1], Vuppu Vinay Pradeep[1], Shruti Singh[1], and Rajadurai Chandrasekar[1, ✉]

[1]Advanced Photonic Materials and Technology Laboratory
School of Chemistry and Centre for Nanotechnology
University of Hyderabad, Prof. C. R. Rao Road, Gachibowli, Hyderabad 500 046, Telangana, India
E-mail: r.chandrasekar@uohyd.ac.in

Supplementary Methods

1. Synthesis and Crystal growth

a) Synthesis of BFIMP: 2-bromo-5-fluoroaniline (190 mg, 1 mmol) and 3,5-dibromosalicyldehyde (280 mg, 1 mmol) were taken in methanol and sonicated for 5-10 minutes. The reaction was monitored using TLC. The obtained compound was recrystallized using MeOH to obtain millimeter-long crystals. Melting Point: 151-153°C. ^{1}H NMR (500 MHz, $CDCl_3$): δ 13.70 (s, 1H), 8.52 (s, 1H), 7.82 (d, J = 2.25 Hz, 1H), 7.67 (m, 1H), 7.5 (d, J = 2.3 Hz, 1H), 7.02 (m, 1H), 6.97 (m, 1H); ^{13}C NMR (125 MHz, $CDCl_3$): δ 163.97, 162.28, 157.63, 139.57, 134.92, 134.43, 120.90, 116.51, 116.33, 112.91, 111.16, 107.19, 106.99.

b) Preparation of millimetre-sized crystals of BFIMP: The bulk BFIMP crystals were obtained by dissolving BFIMP compound in ethyl acetate or methanol. Later, the solution was slightly heated and left aside for crystallization for a few days. After the formation of the crystals, one of the crystals from the mother liquor was taken out and mounted for single-crystal X-ray diffraction analysis.

c) Preparation of microcrystals of BFIMP: For self-assembly, BFIMP (1 mg) was dissolved in ethyl acetate (2 mL) in a clean vial. Then, 2-3 drops of the solution were drop casted onto a clean glass coverslip and left for slow evaporation. Once the solvent was evaporated, it resulted in twisted-shaped long and branched microcrystals (See Movie 3).

2. Materials

All chemicals and solvents (2-bromo-5-fluoroaniline, 3,5-dibromosalicyldehyde, methanol and ethylacatate) were purchased from commercial sources (TCI chemicals, Sigma Aldrich and Merck). HPLC solvents with 99.7% purity were used for synthesis and self-assembly.

3. Instrumental Methods

a) NMR spectroscopy: ^{1}H and ^{13}C NMR spectra were recorded on a Bruker DPX 500 MHz spectrometer with a solvent proton as internal standard ($CDCl_3$: ^{1}H: 7.26 ppm, ^{13}C: 77.16 ppm).

Commercially available deuterated $CDCl_3$ was used. Chemical shifts (δ) are given in parts per million (ppm). Spectra were processed using topspin 4. 1. 1. software.

b) Optical absorbance and emission studies: The measurements were done on Jasco V-750 spectrophotometer in a diffuse reflectance UV−visible (DR−UV−vis) mode. The reflectance spectra were converted to an absorbance using the Kubelka−Munk function. The solid-state emission spectra were collected using FP-8500 fluorescence spectrometer. The parameters used where excitation and emission bandwidth are 2.5 nm, Response=1sec, Sensitivity= Medium, Data interval=0.5nm, Scan speed 500 nm/min.

c) Single crystal X-ray diffraction: Single-crystal X-ray diffraction data was collected on Rigaku Oxford XtaLAB ProPilatus3 R 200K-A detector system equipped with a CuKα, MicroMax-003 microfocus sealed tube operated at 50 kV and 0.6 mA. All data were collected at 298 K, and the data reduction was performed using CrysAlisPro software. The crystal structure was refined and solved by using the OLEX software. Face indexing was done using CrysAlisPro software.

d) Confocal Micro-spectroscopy studies:

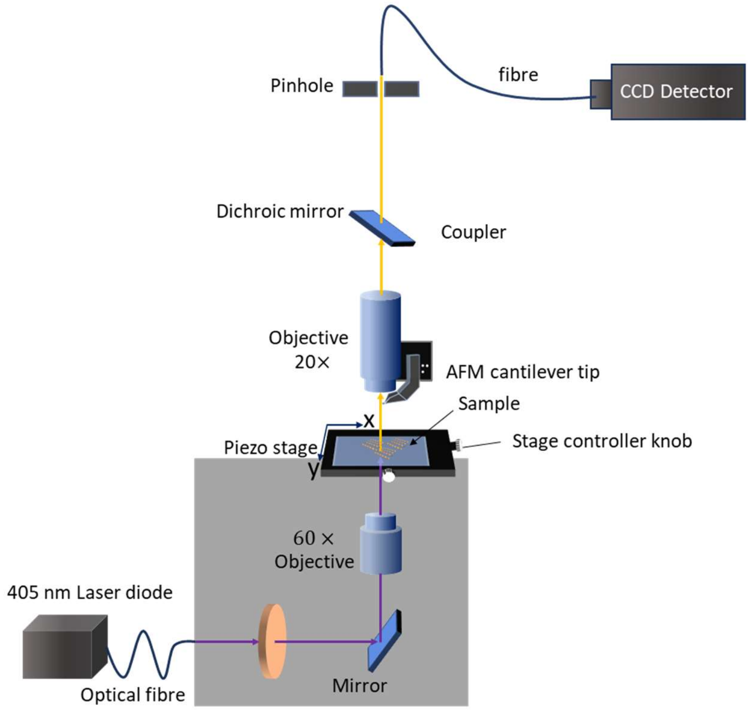

Supplementary Figure 1. Transmission mode-Confocal microscopy set-up

The optical experiments of a single microcrystal were carried out on a transmission mode setup of the Wi-Tec alpha 300 AR laser confocal optical microscope (LCOM) equipped with a Peltier-cooled CCD detector. Using 300 grooves/mm grating BLZ = 750 nm, the accumulation time was adjusted to 30 s and integration time was typically made 0.5 s. Each single spectrum is obtained by ten averaged accumulations. A diode 405 nm laser source was used for excitation. 20×, 60× and 150× objectives

were used for spectra and image collection, respectively. The power used for the excitation of microcrystals was 0.05 mW. All the experiments were carried out under ambient conditions.

e) Micromanipulation of the crystals: The micromanipulation experiments were performed manually using the AFM facility attached to the above-mentioned confocal microscope setup. Single microcrystals of BFIMP were selected and isolated from the bunch of microcrystals. Then the microcrystals were bent, rolled, stacked, made to stand, interlocked, and transferred onto the coverslip (borosilicate; Borosil), using an AFM cantilever tip. An AFM cantilever (TipsNano: NSG10, force constant 3.1− 37.6 N/m) was used for the mechanical manipulation.

Supplementary Notes:

The details of the various micromanipulation operations performed on twisted crystals are given below (Also see the representative **Supplementary movies**):

(i) For a typical crystal bending experiment [Movie 4], the substrate containing the microcrystals was placed on the piezo stage. Later, the AFM-tip height was adjusted in the -z direction (perpendicular to the substrate; -sign indicates tip movements downwards) in such a way that tip could make contact with the microcrystal kept on the substrate. At this constant cantilever height, the piezo stage was carefully moved in x and/or y directions to bring mechanical deformation in the microcrystal through the tip by making crystal-tip contact.

(ii) For a typical crystal cutting experiment, the substrate containing the long microcrystal was placed on the piezo stage. Later, the AFM-tip height was adjusted in the -z direction (perpendicular to the substrate) in such a way that tip make contact (away from the termini) with the selected flat surface of the microcrystal kept on the substrate. Subsequently, the AFM-tip was moved downwards in the -z direction to apply pointed force on the contact region resulting in the cutting of the crystal.

(iii) For the crystal leaning/standing experiment [Movie 5], the substrate containing the microcrystals was placed on the piezo stage. Later, the AFM-tip height was adjusted in the -z direction (perpendicular to the substrate) in such a way that tip could make contact with the microcrystal’s one of the termini (Note: there is a gap between the twisted crystal termini and substrate). By pressing one of the termini of the microcrystal with the cantilever tip towards the substrate, the opposite terminal was lifted in a slanted position. Later, the cantilever was lifted up and the crystal remained in the slanted position without any support. By adjusting the piezo stage in x and/or y directions and cantilever height from the substrate, the cantilever was placed below slanted crystals. By moving the cantilever upwards gently at different heights, slanted crystal configurations at various degrees and near vertical configurations were realized.

(iv) For the 3D stacking of twisted microcrystals experiment [Movie 6], the cut crystals were aligned one by one parallel to each other. For example, initially, by adjusting the AFM-tip height in the z-direction (perpendicular to the substrate) in such a way that tip can make contact with the microcrystal kept on the substrate. Later, at this constant cantilever height, the piezo stage was carefully moved in x and/or y directions to exert mechanical force through the AFM-tip to the crystal to move it. In this way, each crystal was aligned parallel to one another. For the second layer, lifting and dropping operations are involved. The stage was moved in such a way that the tip made contact and attached

with one of the termini of the crystal. The attached crystal was lifted up using cantilever +z movement and orientationally aligned 90° to the first layer and dropped. A similar procedure was followed to make 3rd layer.

(v) For the interlocking of two twisted crystals [Movie 7], initially, AFM-tip height in the z-direction (perpendicular to the substrate) was adjusted in such a way that tip can make contact with the microcrystal kept on the substrate. Then, the piezo stage was carefully moved in x and/or y directions to apply AFM tip force on each crystal to align them axially, in a head-to-head pointed manner in such a way that the dark contrast region of one crystal faces the bright contrast region of another crystal. Later, the piezo stage was carefully moved in +y direction (meaning AFM tip in the -y direction with reference to crystal) to apply force directly on the (100) plane and then in the +x direction (meaning AFM tip in the -x direction with reference to crystal) i.e. the side of the terminal to entwine the two crystals into a single unit.

f) Field-Emission Scanning Electron Microscopy: The morphological analysis of microcrystals was performed using a Zeiss field-emission scanning electron microscope (FESEM) operating at 3 kV. All the experiments were performed after gold coating.

g) Fluorescence Lifetime Imaging Microscopy: PL lifetime images were recorded on a time-resolved (Micro-Time 200, Pico Quant) confocal PLIM setup equipped with an inverted microscope (Olympus IX 71). The microcrystals were illuminated by a 405 nm ps diode pulse laser (power ≈5 μw) with a stable repetition rate of 20 MHz (FWHM: 176 ps) through a water immersion objective (Olympus UPlans Apo; 60×; NA 1.2). The signal from the samples was collected by the same objective and passed through the dichroic mirror, filtered by using a 430 nm long-pass filter to cut off any excitation light. The signal was then focused onto a 50 μm diameter pinhole to remove the out-of-focus signal, recollimated, and directed onto a (50/50) beam splitter before entering two single-photon avalanche photodiodes. The data acquisition was carried out with a SymPhoTime software-controlled PicoHarp 300 time-correlated single-photon counting module in a time-tagged time-resolved mode. The overall resolution of the setup was 4 ps.

h) Transmission electron microscopy: The microstructures were probed for their detailed morphology and crystalline nature using a multi-purpose JEOL F200 transmission electron microscope (TEM) operating at 200 kV acceleration voltage. The micro/nanocrystals were obtained by drop-casting a drop of BFIMP solution (1 mg / 2 mL in ethyl acetate) on a TEM copper grid. The selected area electron diffraction dot pattern analysis was carried out using the ImageJ software and diffraction pattern in XRD.

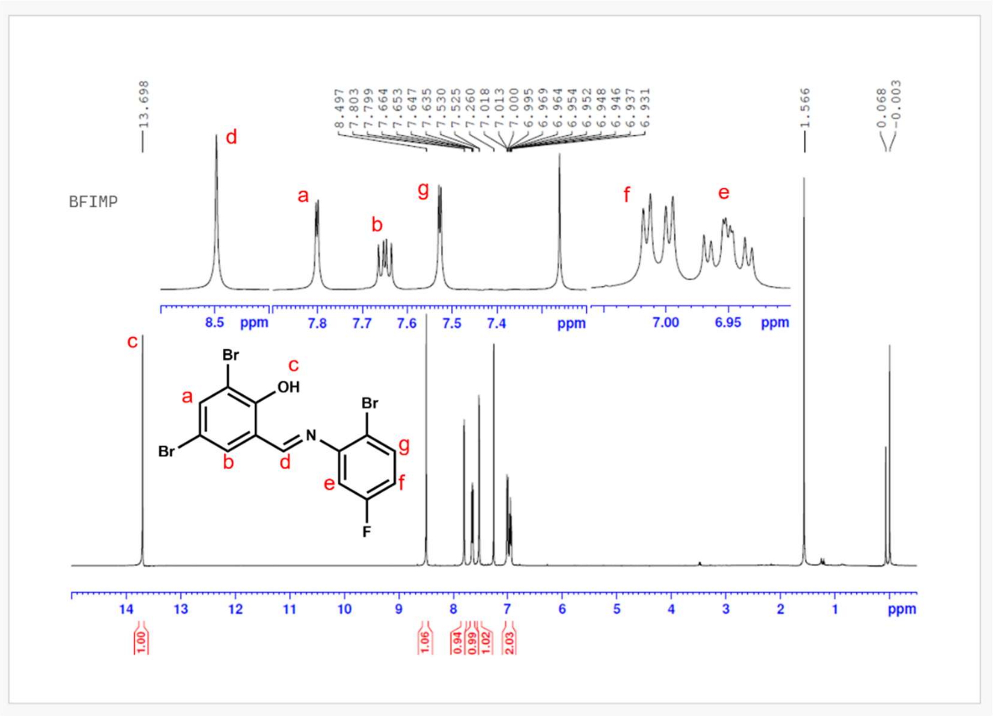

Supplementary Figure 2. NMR Data. ^{1}H NMR spectrum (500 MHz) of BFIMP in $CDCl_3$.

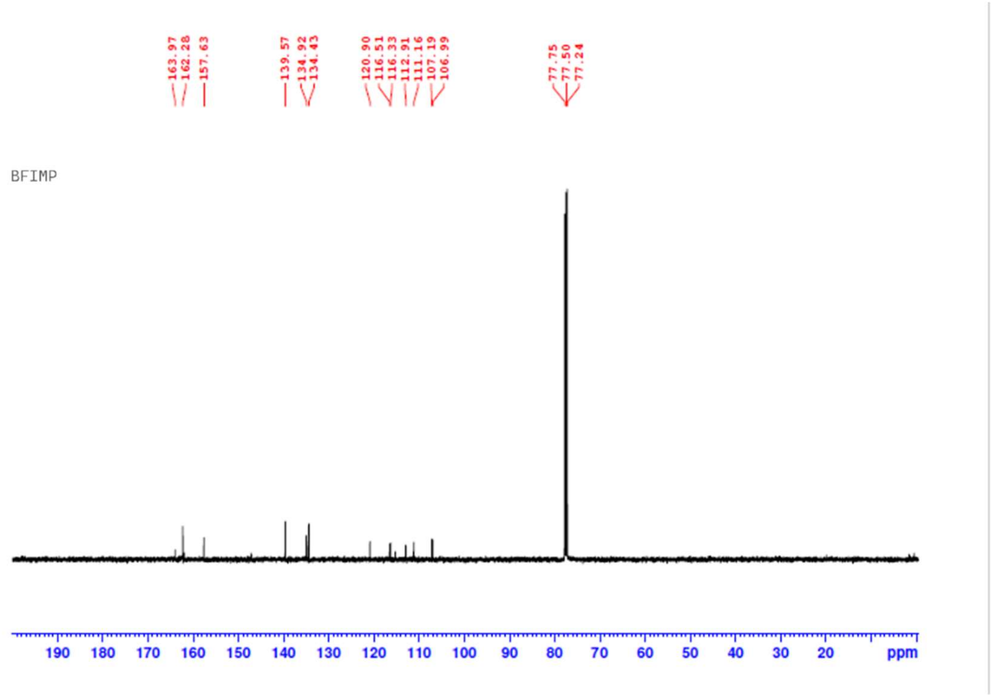

Supplementary Figure 3. NMR data. ^{13}C NMR spectrum of BFIMP in $CDCl_3$.

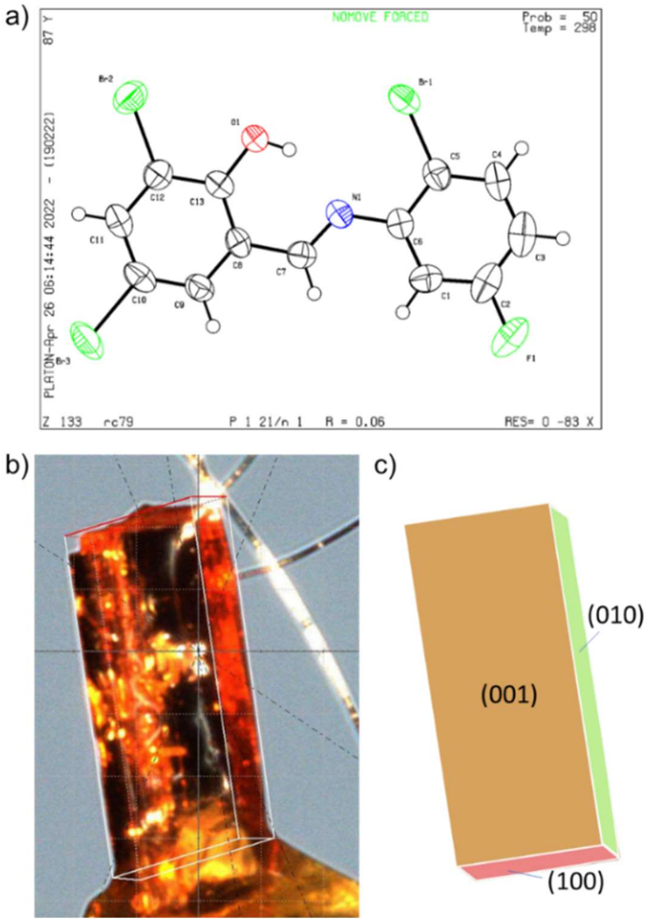

Supplementary Figure 4. Single-crystal X-ray data. a) ORTEP view of BFIMP with thermal ellipsoids shown at 50% probability. b) Picture of the mounted BFIMP crystal and c) the graphical representation of indexed planes of BFIMP.

Supplementary Table 1. Crystallographic data of BFIMP single-crystal.

Compound Name	BFIMP
CCDC number	2300243
Empirical formula	$C_{13}H_7Br_3FNO$
Formula weight	451.93
Temperature (K)	298
Wavelength	0.71073 Å
Crystal system	Monoclinic
Space group	P21/n
Crystal color	Orange
Cell Lengths (Å)	**a** = 6.9261(3), **b** = 12.6133(5), **c** = 16.3232(7)
Cell Angle (°)	**α** = 90, **β** = 100.975(4), **γ** = 90
Cell Volume (Å^3)	1399.93(10)
Density (g/cm^3)	2.144
Z	4
R-factor (%)	6.48

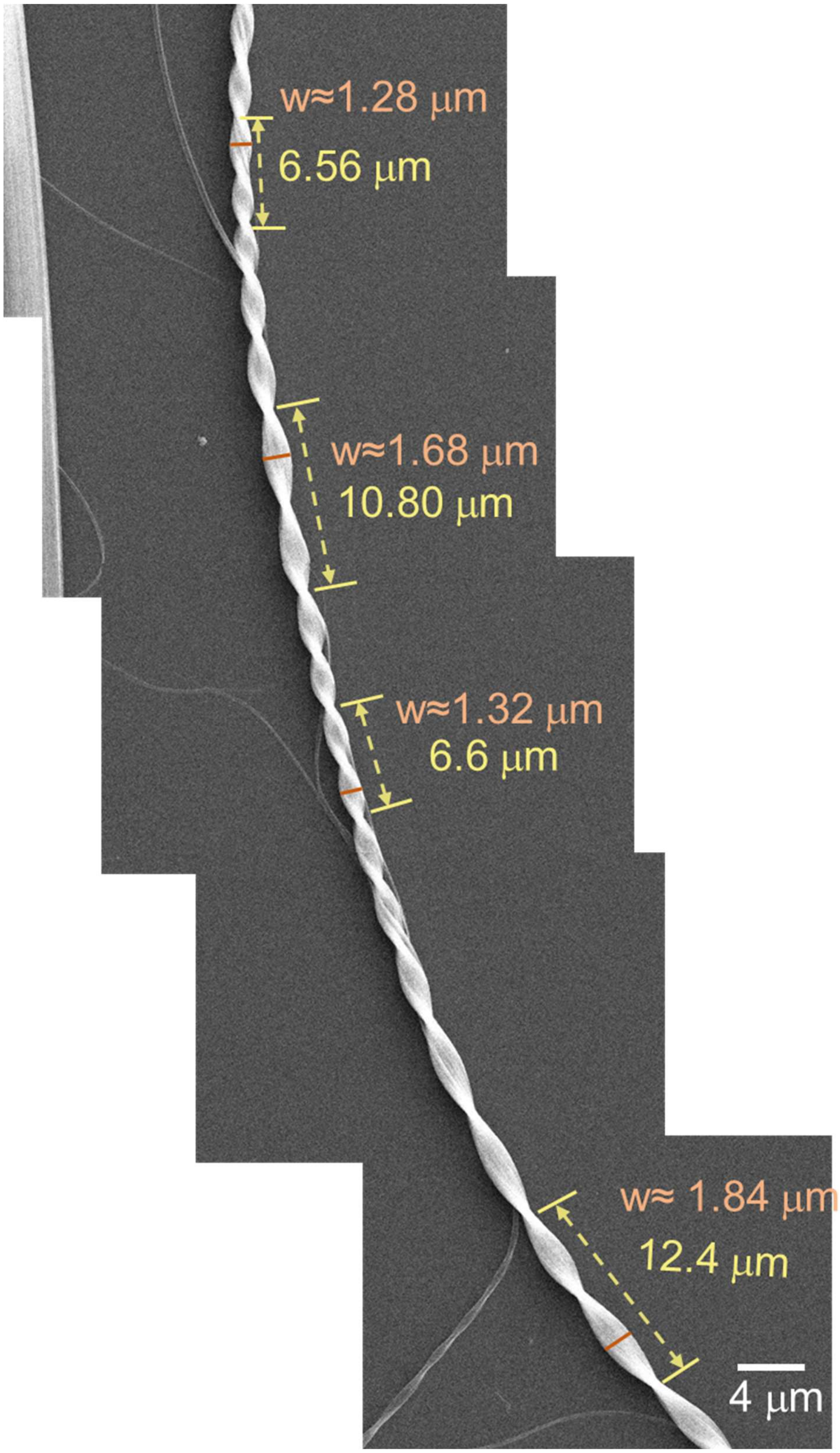

Supplementary Figure 5. A high aspect ratio single twisted crystal. Stitched FESEM images of a high aspect ratio twisted crystal display the variation of pitch length within the same crystal.

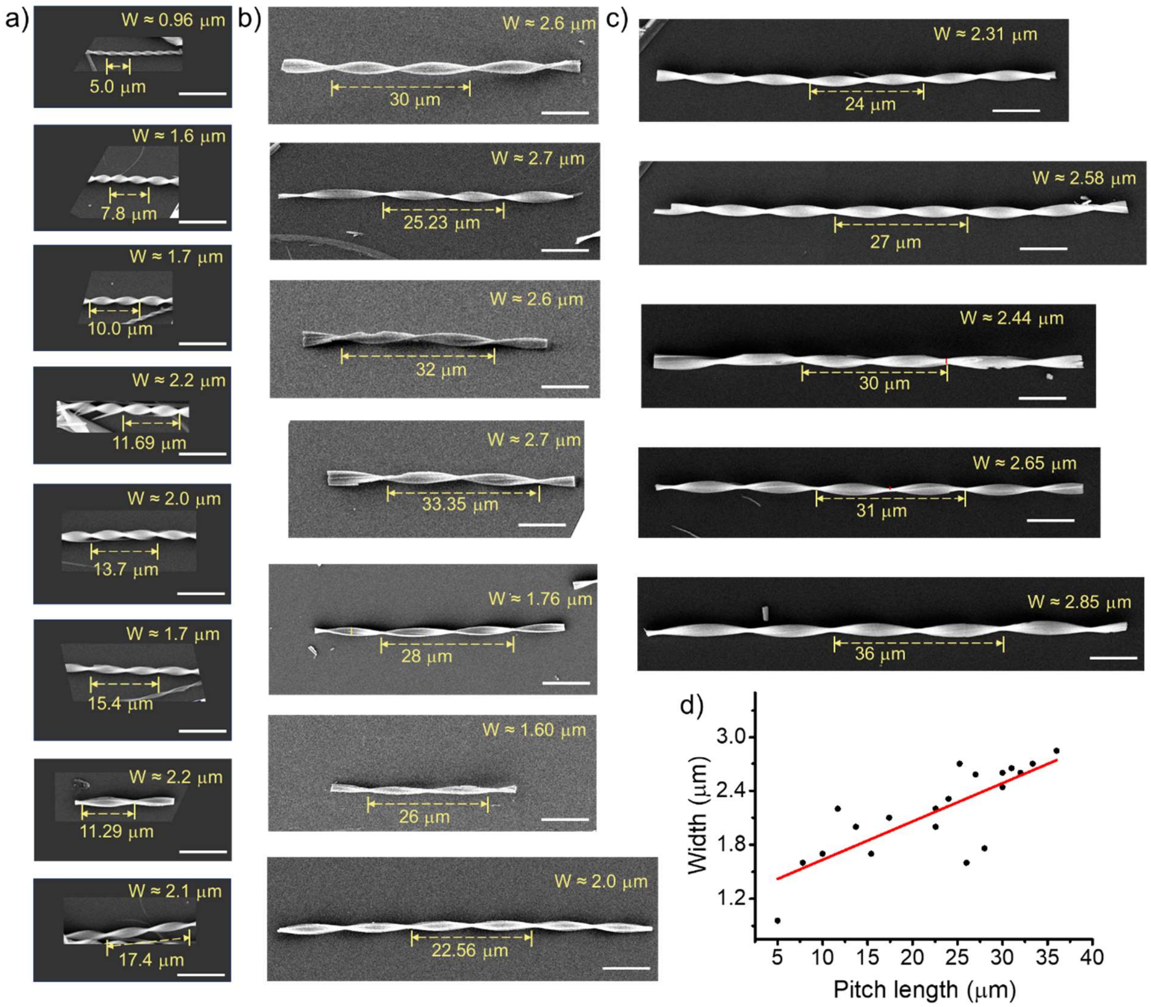

Supplementary Figure 6. Aspect ratio and pitches in representative twisted crystals. a) FESEM images (presented on a black rectangular background panel) of a series of twisted crystals with varying twist periods (pitch lengths). b) Plot of the width of the crystal with their respective pitch lengths. This experiment was independently performed for several times. See also **Figure 3**, and **Supplementary Figures 7 and 22.**

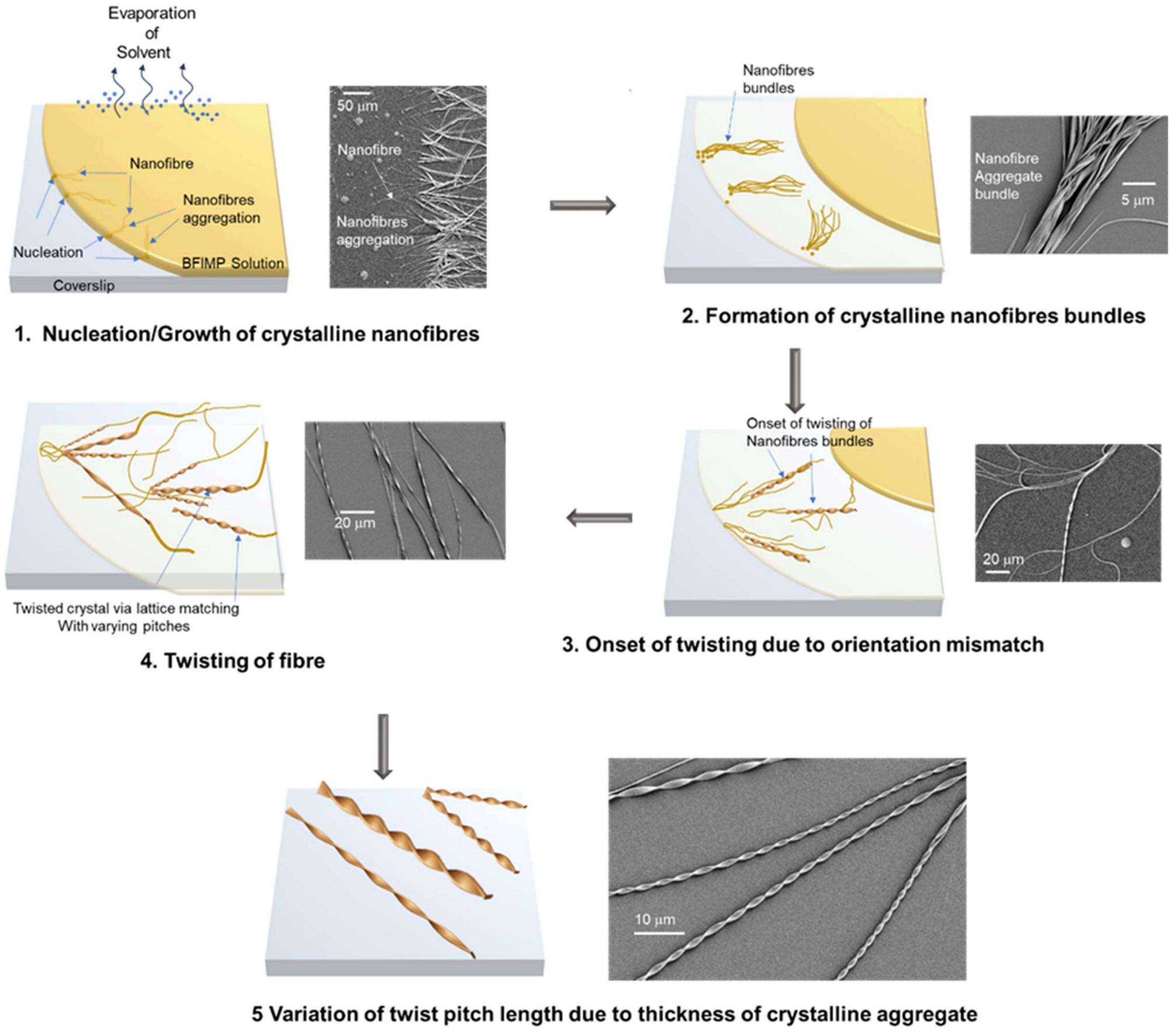

Supplementary Figure 7. **Growth mechanism of twisted crystal.** Graphical representation showing the growth of several orientationally mismatched entwined nanofibres, their cooperative interaction and the associated interfacial strain causing spontaneous twists in growing crystals along with the supporting FESEM images. This experiment was independently performed for several times. Representative videos of two independent experiments are shown in **Supplementary movies 3 and 9.**

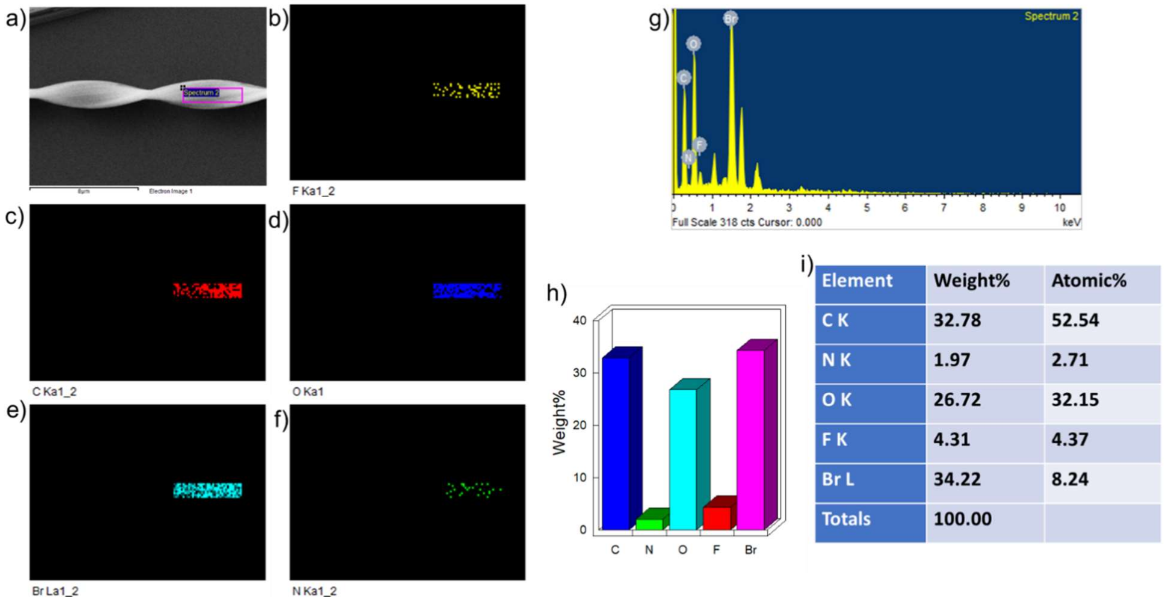

Element	Weight%	Atomic%
C K	32.78	52.54
N K	1.97	2.71
O K	26.72	32.15
F K	4.31	4.37
Br L	34.22	8.24
Totals	100.00	

Supplementary Figure 8. Elemental mapping in a twisted crystal. EDX analysis of twisted microcrystal.

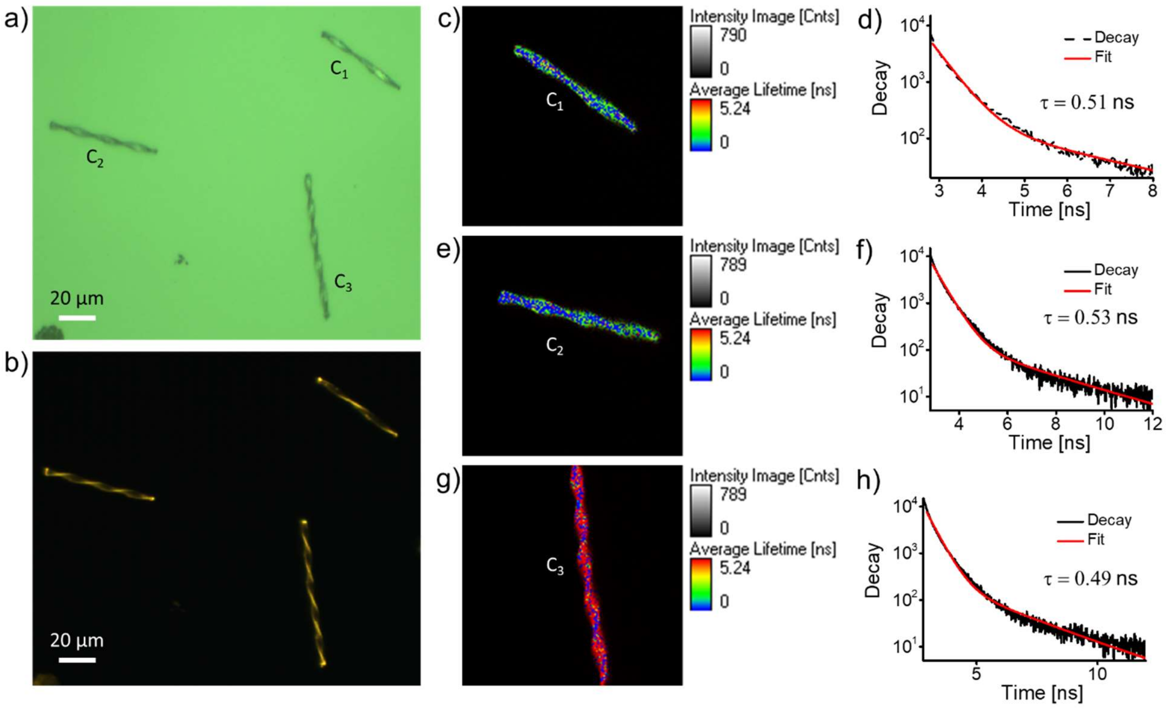

Supplementary Figure 9. FL lifetime image of twisted crystals. a) Confocal and b) its corresponding FL image of twisted microcrystals. c,e,g) FL lifetime image and d,f,h) its corresponding decay plots of twisted microcrystals. This experiment was independently performed for several times. The results of additional independent experiment are given in **Supplementary Figure 25.**

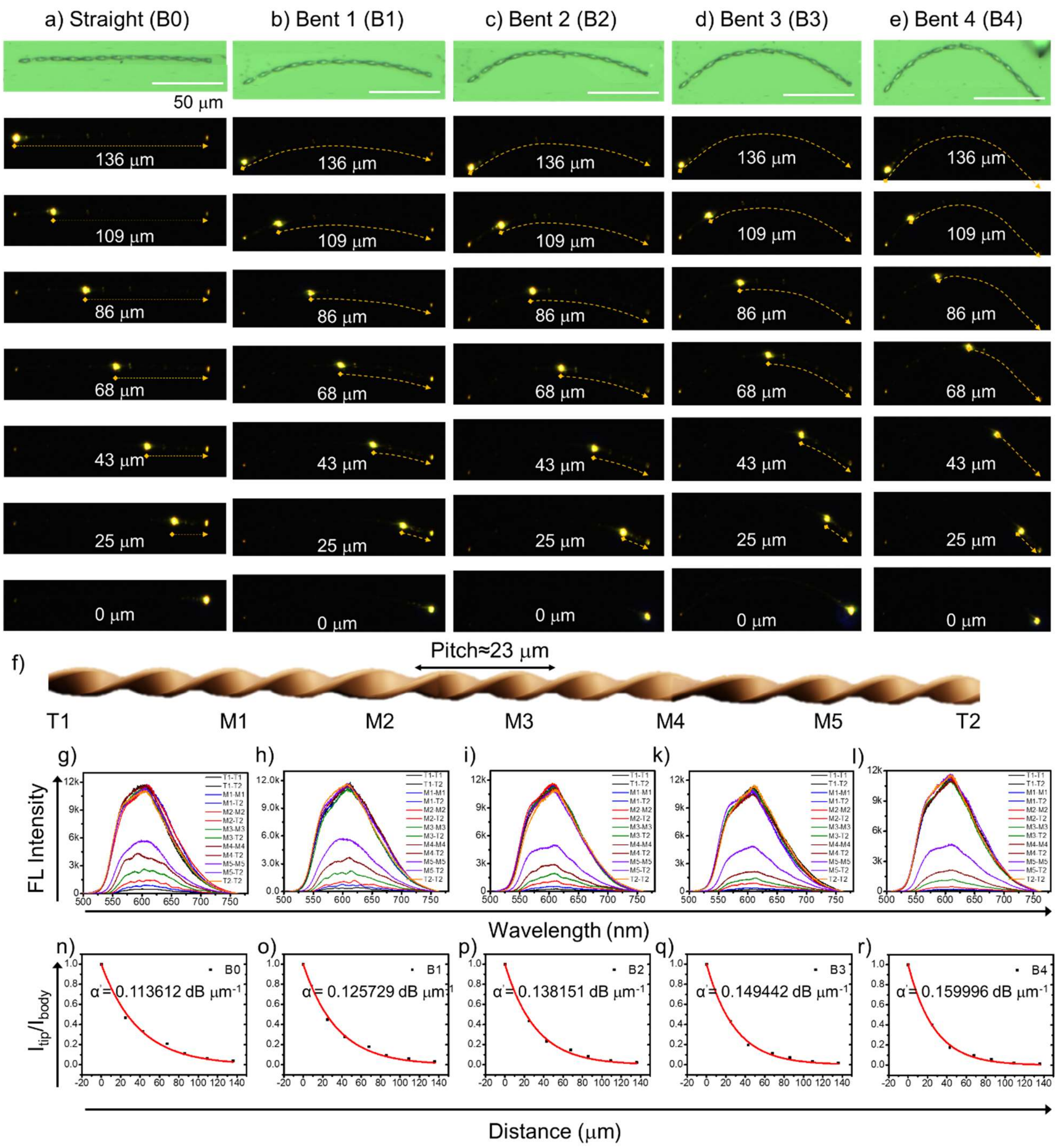

Supplementary Figure 10. Optical waveguiding in straight and bent twisted crystal. Confocal optical and FL images of a) straight and b-e) bent geometries of twisted crystals (B1-B4) exciting with 405 nm laser at different positions (T1, M1-M5, T2). f) Graphical representation of twisted crystal. g-l) FL spectra of corresponding excitation position-dependent waveguiding for a) straight and b-e) bent geometries (B1-B4), respectively. n-r) A plot of the I_{tip}/I_{body} versus the distance of the light propagation path used to estimate the optical loss coefficient (α') for B0-B4 twisted waveguides.

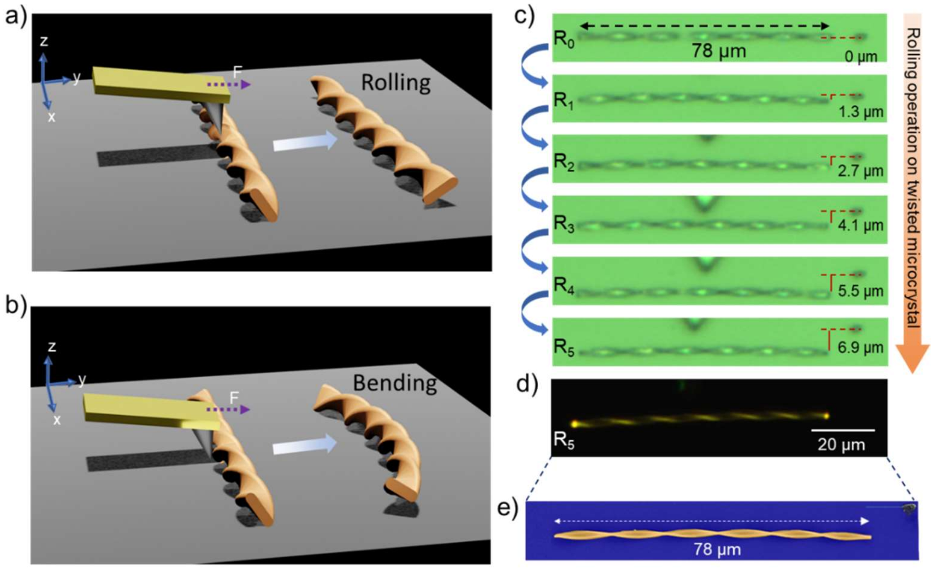

Supplementary Figure 11. Bending and rolling twisted crystal. Graphic representing the a) bending and b) rolling locomotion of twisted microcrystals using AFM cantilever tip. The point of contact between the cantilever tip and the microcrystal decides the operation (bending/rolling) of the microcrystal. c) Sequential confocal images showing the rolling locomotion of twisted microcrystal. d) The corresponding FL and e) color-coded FESEM image.

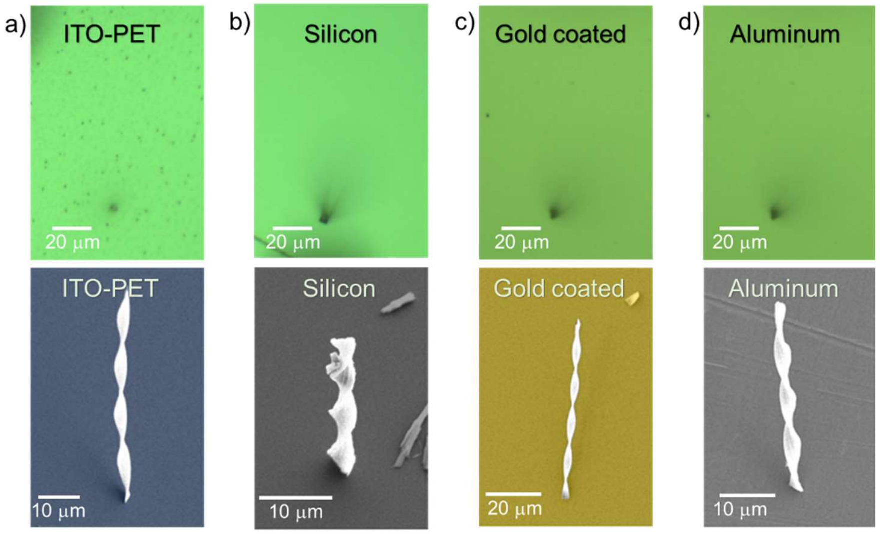

Supplementary Figure 12. Standing twisted crystal at various substrates. The confocal microscopy and color-coded FESEM images showing the vertical standing of twisted microcrystals on a) ITO-PET, b) silicon, c) gold-coated glass and d) aluminium substrates. The experiment was repeated several times.

Note: The crystals were grown on a cover slip, and the long crystal were cut and transferred to different substrates using lifting and dropping mechanical micromanipulation using AFM cantilever tip.

Additional independent experiments are shown in **Supplementary Figure 23.**

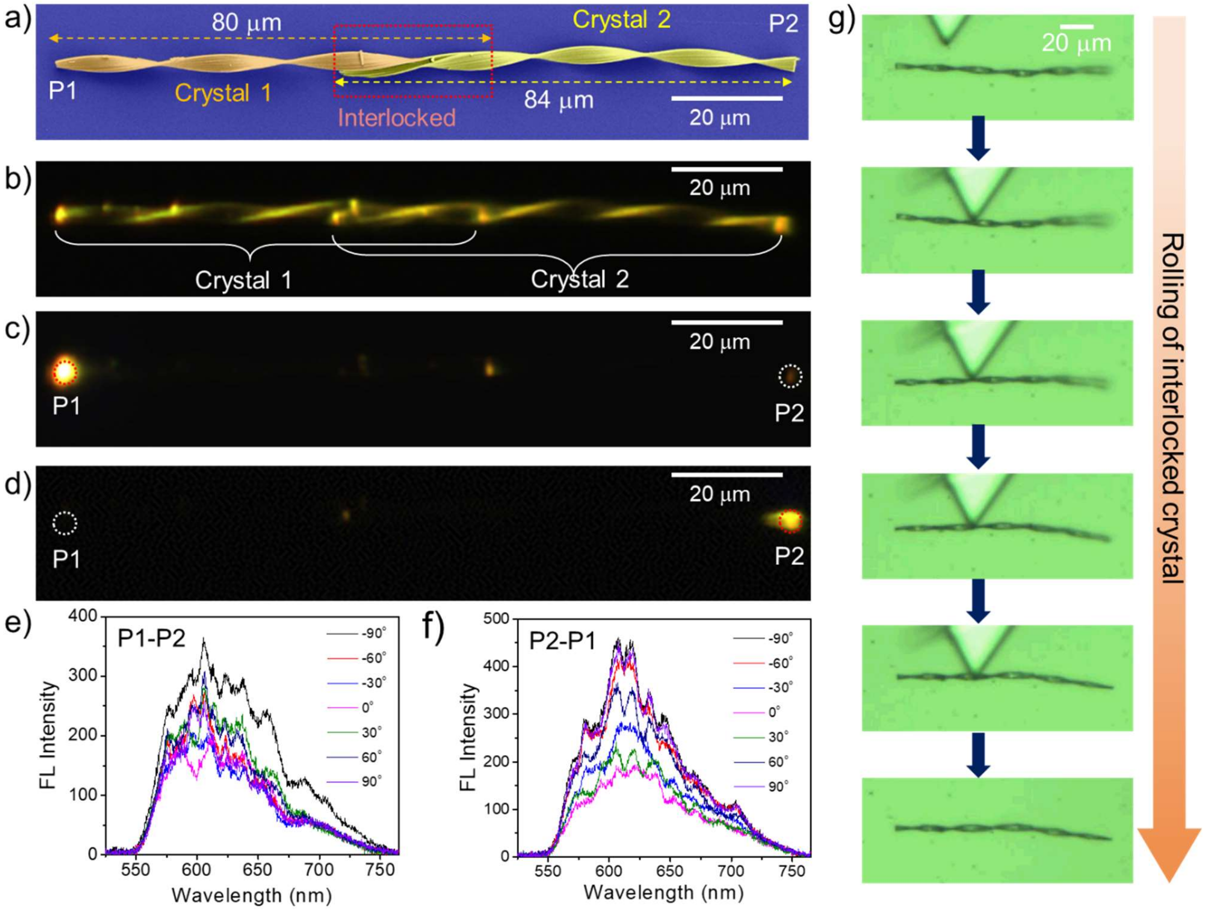

Supplementary Figure 13. Polarization rotation studies in interlocked twisted crystal waveguides. a) FESEM and b) FL image of a twisted interlocked crystal of BFIMP. FL image of crystal excited at c) P1 and d) P2 and recording the FL spectra at e) P2 and f) P1, respectively. g) Sequence of optical images taken while rolling the interlocked crystal with AFM cantilever tip.

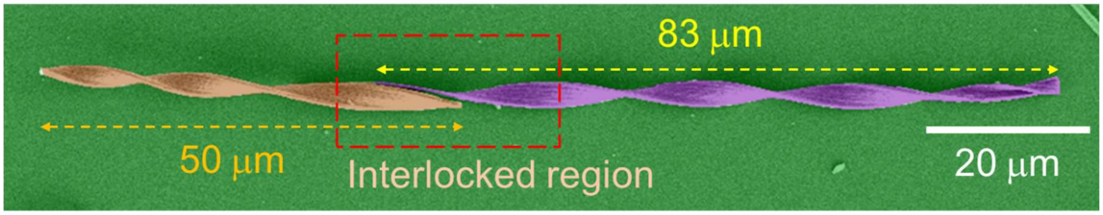

Supplementary Figure 14. Interlocked twisted crystals. Color-coded FESEM image of axially interlocked twisted crystals of lengths 50 μm and 83 μm.

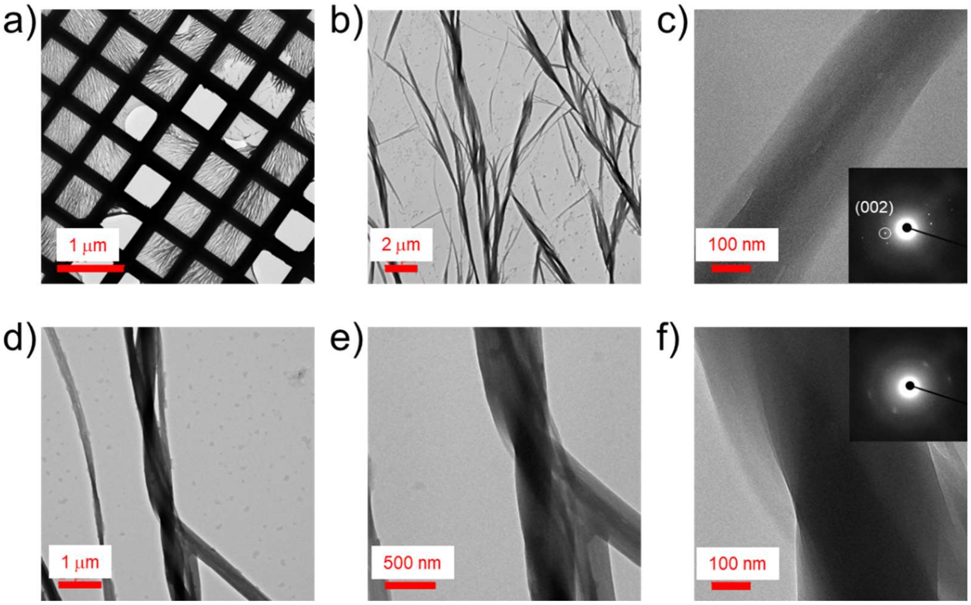

Supplementary Figure 15. Electron microscopy studies of twisted crystals. a-f) The TEM images and the SAED pattern of the as-grown microcrystals.

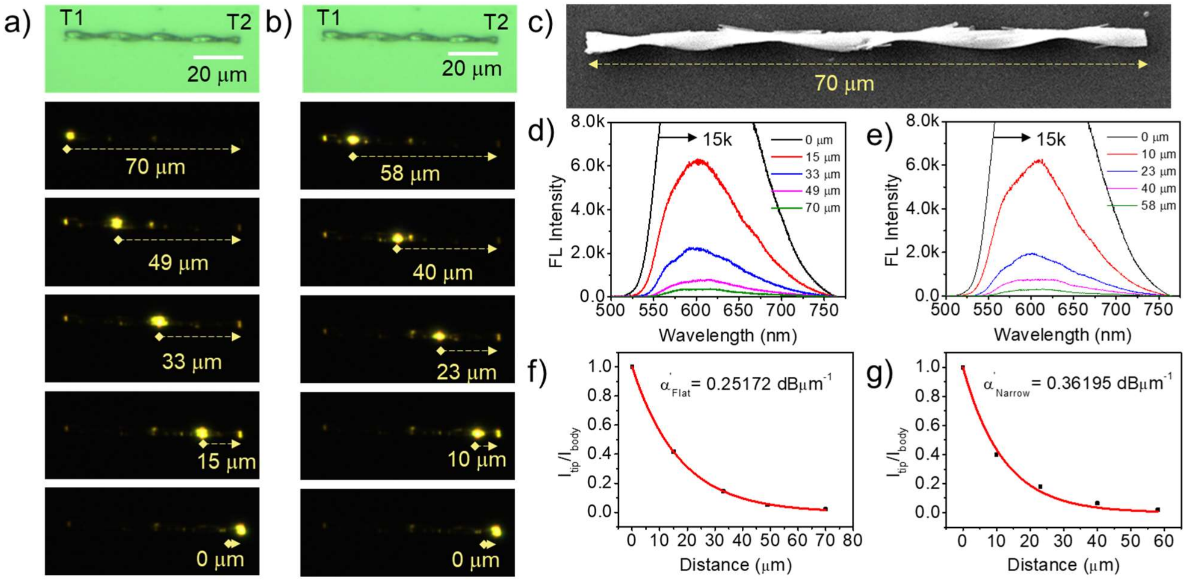

Supplementary Figure 16. Optical waveguiding studies in twisted crystals. Confocal optical and FL image of straight twisted crystal exciting with 405 nm laser at a) flat positions only and b) narrow positions only. c) FESEM image of a twisted crystal. d,e) Excitation position-dependent waveguiding: FL spectra recorded at T2 for excitations at flat and narrow positions, respectively. f,g) A plot of the I_{tip}/I_{body} versus the distance of the light propagation path used to estimate the optical loss coefficient (α') while exciting flat and narrow positions, respectively. I_{body} = FL intensity at T1.

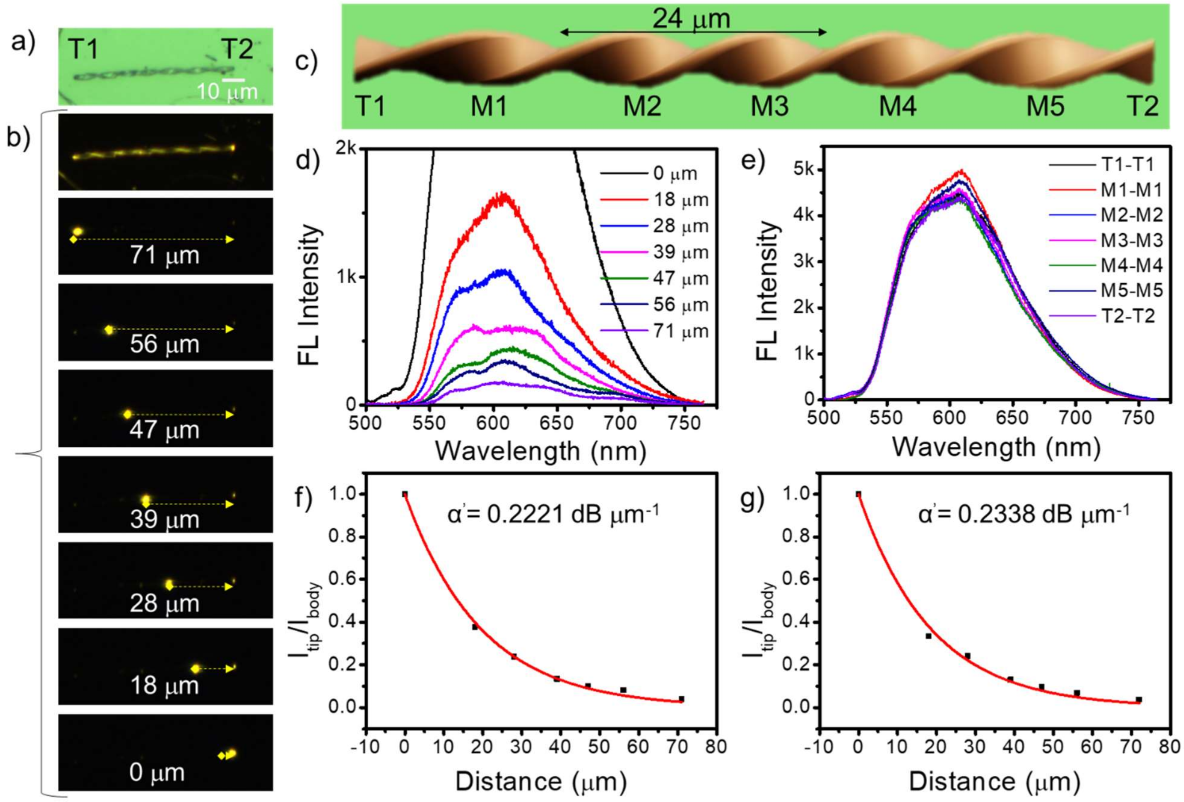

Supplementary Figure 17. Optical waveguiding studies in twisted crystals with varying aspect ratios. a) Confocal optical and b) FL images of straight twisted crystal with P=24 μm excited with a 405 nm laser at different positions along the crystal long axis used for the estimation of optical loss (α') using $I_{tip}/I_{body} = e^{-\alpha' D}$. c) Graphical representation of twisted crystal with a P=24 μm with excitation/collection position labels. d) Excitation position-dependent FL spectra of waveguiding crystal when I_{body} is FL intensity at T1 and I_{tip} is FL intensity at T2. e) Excitation position-dependent FL spectra of the waveguiding crystal when I_{body} is FL intensity at each excitation position (T1, M1-M5 and T2) and I_{tip} is FL at T2 f,g) The plots of I_{tip}/I_{body} versus the distance of the light propagation (D) used for the estimation of α' for crystals using the spectra shown in d) and e), respectively.

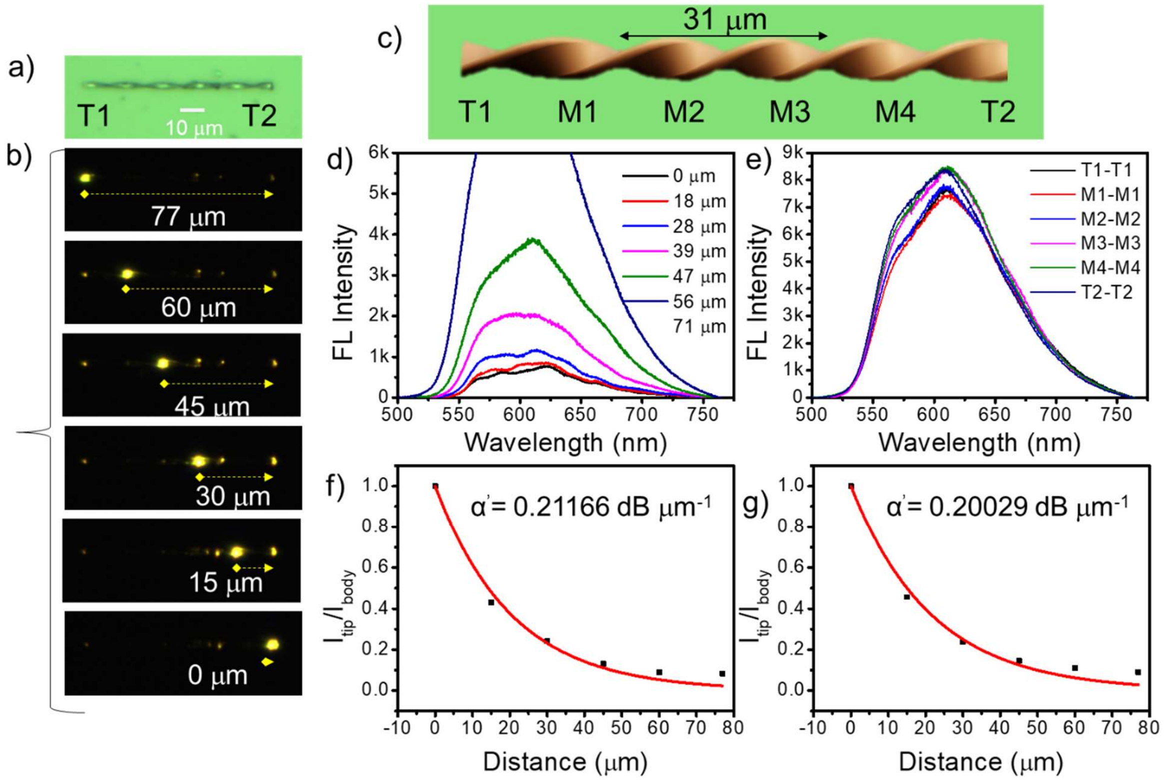

Supplementary Figure 18. Optical waveguiding studies in twisted crystals with varying aspect ratios. a) Confocal optical and b) FL images of straight twisted crystal with P=31 μm excited with a 405 nm laser at different positions along the crystal long axis used for the estimation of optical loss (α') using $I_{tip}/I_{body} = e^{-\alpha' D}$. c) Graphical representation of twisted crystal with a P=31 μm with excitation/collection position labels. d) Excitation position-dependent FL spectra of waveguiding crystal when I_{body} is FL intensity at T1 and I_{tip} is FL intensity at T2. e) Excitation position-dependent FL spectra of the waveguiding crystal when I_{body} is FL intensity at each excitation position (T1, M1-M5 and T2) and I_{tip} is FL intensity at T2 f,g) The plots of I_{tip}/I_{body} versus the distance of the light propagation (D) used for the estimation of α' for crystals using the spectra shown in d) and e), respectively.

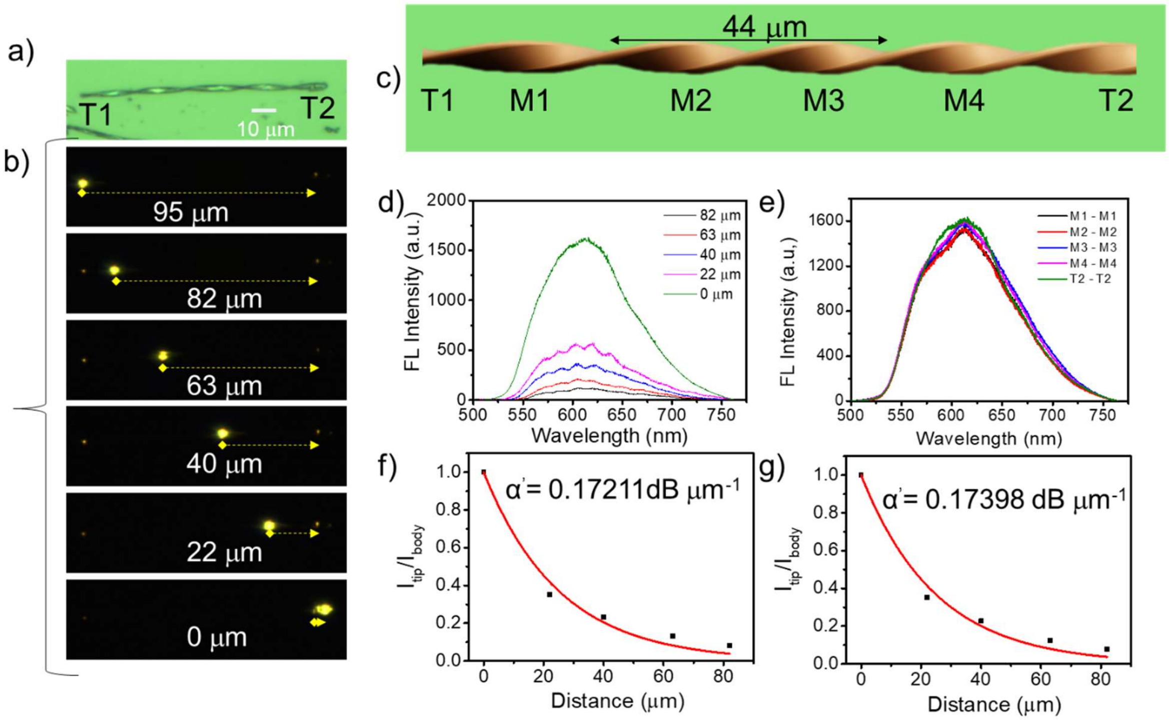

Supplementary Figure 19. Optical waveguiding studies in twisted crystals with varying aspect ratios. a) Confocal optical and b) FL images of straight twisted crystal with P=44 μm excited with a 405 nm laser at different positions along the crystal long axis used for the estimation of optical loss (α') using $I_{tip}/I_{body} = e^{-\alpha' D}$. c) Graphical representation of twisted crystal with a P=44 μm with excitation/collection position labels. d) Excitation position-dependent FL spectra of waveguiding crystal when I_{body} is FL intensity at T1 and I_{tip} is FL intensity at T2. e) Excitation position-dependent FL spectra of the waveguiding crystal when I_{body} is FL intensity at each excitation position (T1, M1-M5 and T2) and I_{tip} is FL at T2 f,g) The plots of I_{tip}/I_{body} versus the distance of the light propagation (D) used for the estimation of α' for crystals using the spectra shown in d) and e), respectively.

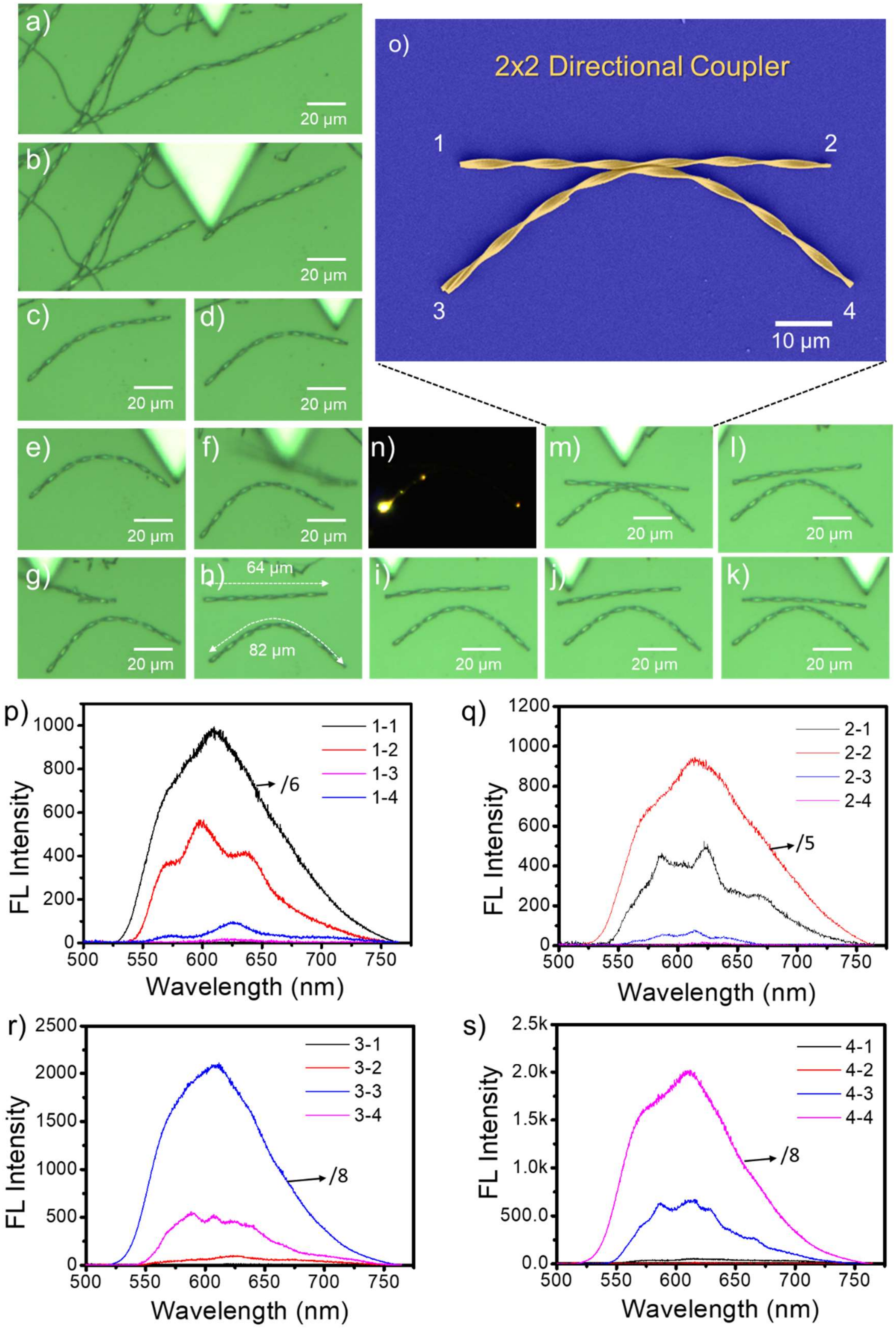

Supplementary Figure 20. Light splitting in a 2x2 twisted crystal directional coupler. a-m) Confocal and n) its corresponding FL image of a twisted directional coupler. o) FESEM image of corresponding 2x2 directional coupler. p-s) FL spectra recorded at terminals 1-4 while exciting with 405nm laser at other terminals, respectively.

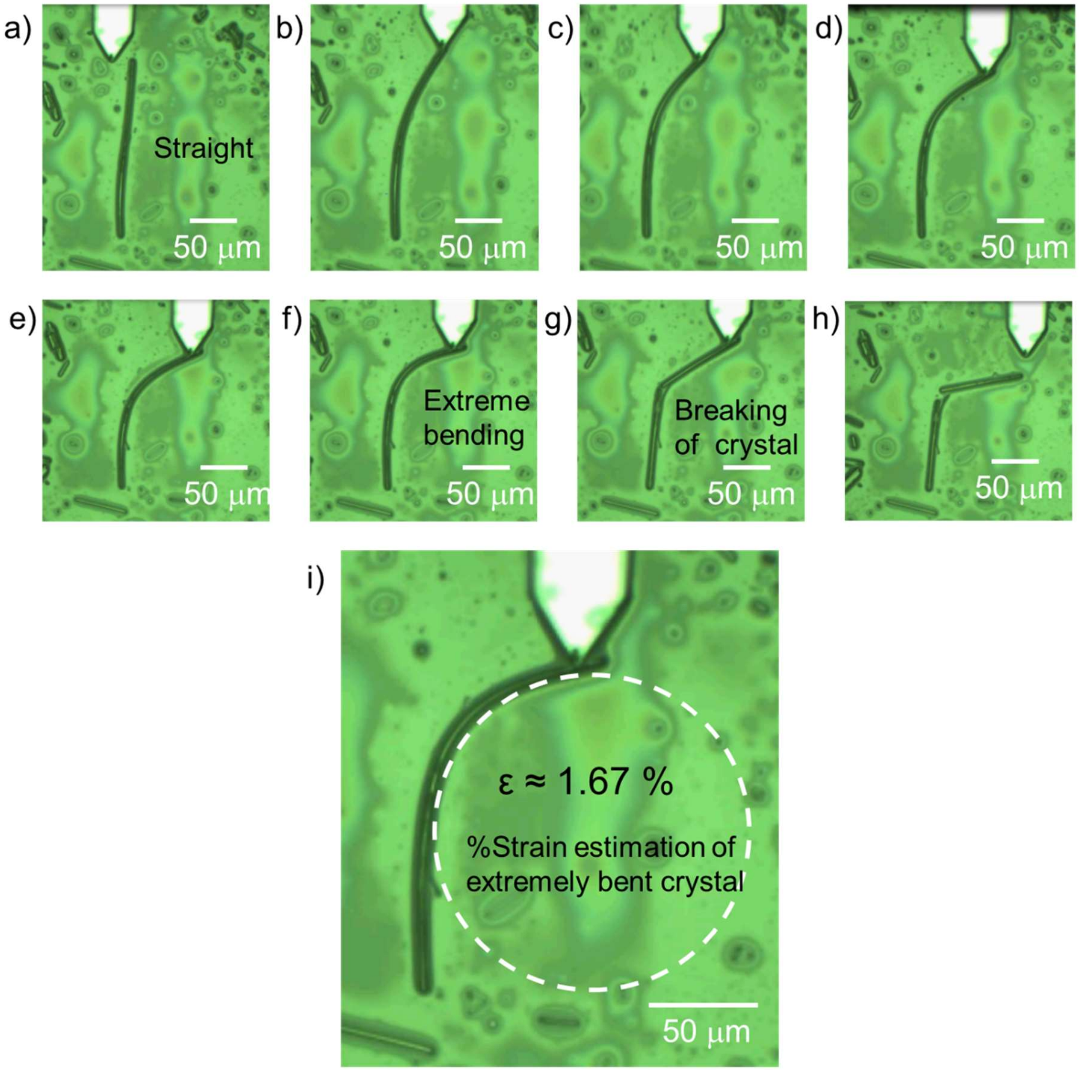

Supplementary Figure 21. Mechanical bending of an untwisted crystal. Confocal microscopy images of BFIMP untwisted crystal in a) straight, b-e) bending of crystal, f) extreme bending, g,h) breaking of crystal (kept on an oil-coated coverslip) after extreme force applied by AFM tip. i) Estimation of strain% of crystal in extremely bent geometry.

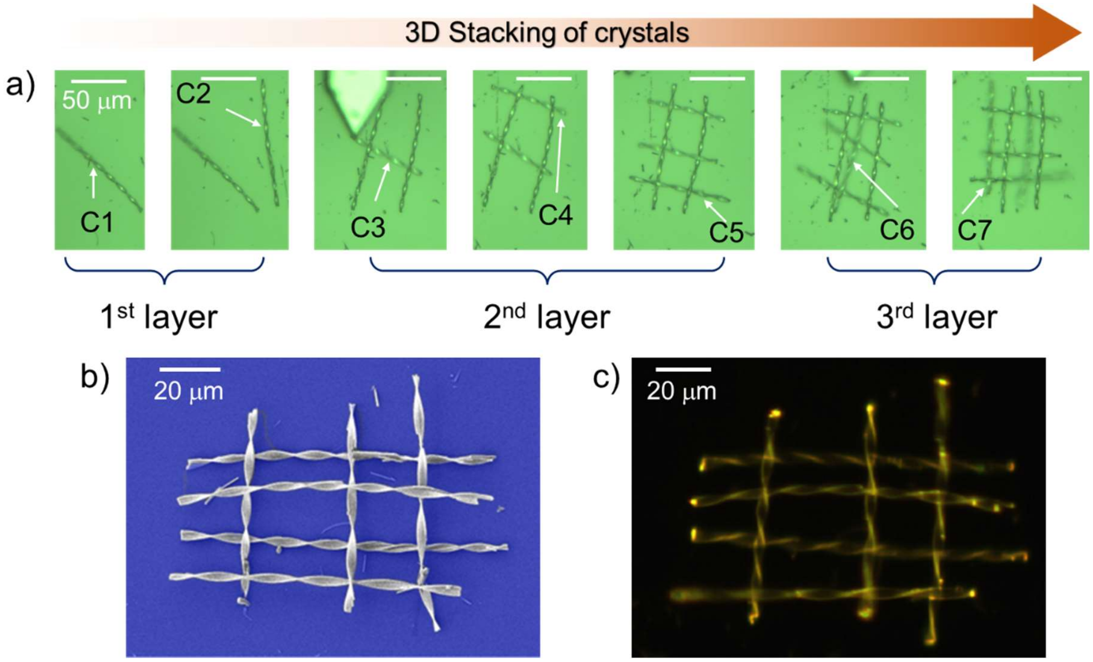

Supplementary Figure 22. 3D stacking of twisted microcrystals. a) The sequential confocal images showing the stacking of twisted microcrystals (crystals C1 to C7) on a glass substrate. b) Color-coded FESEM image of 3-layered stacked microcrystals. c) The corresponding FL image of 3-layered stacked microcrystals.

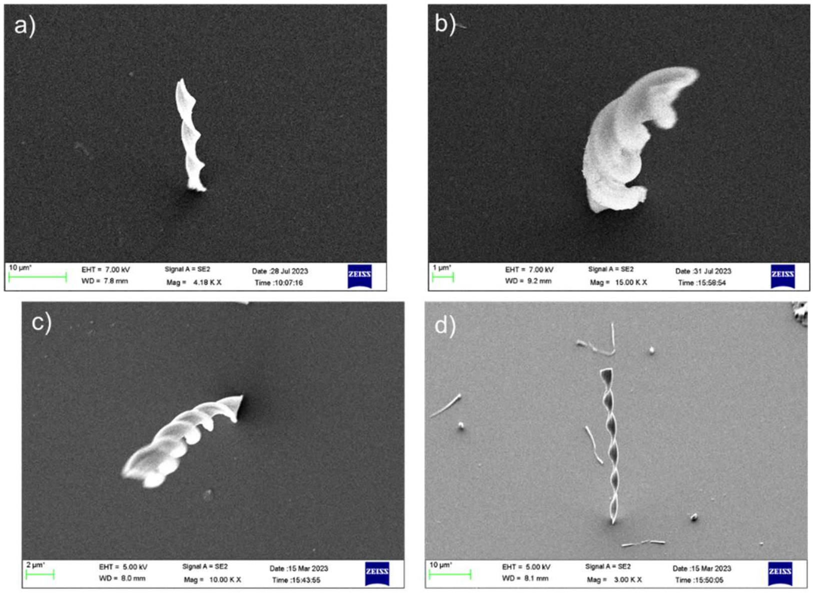

Supplementary Figure 23. Experiments on Standing of twisted crystal on glass substrates. a-e) FESEM images of different twisted standing crystals.

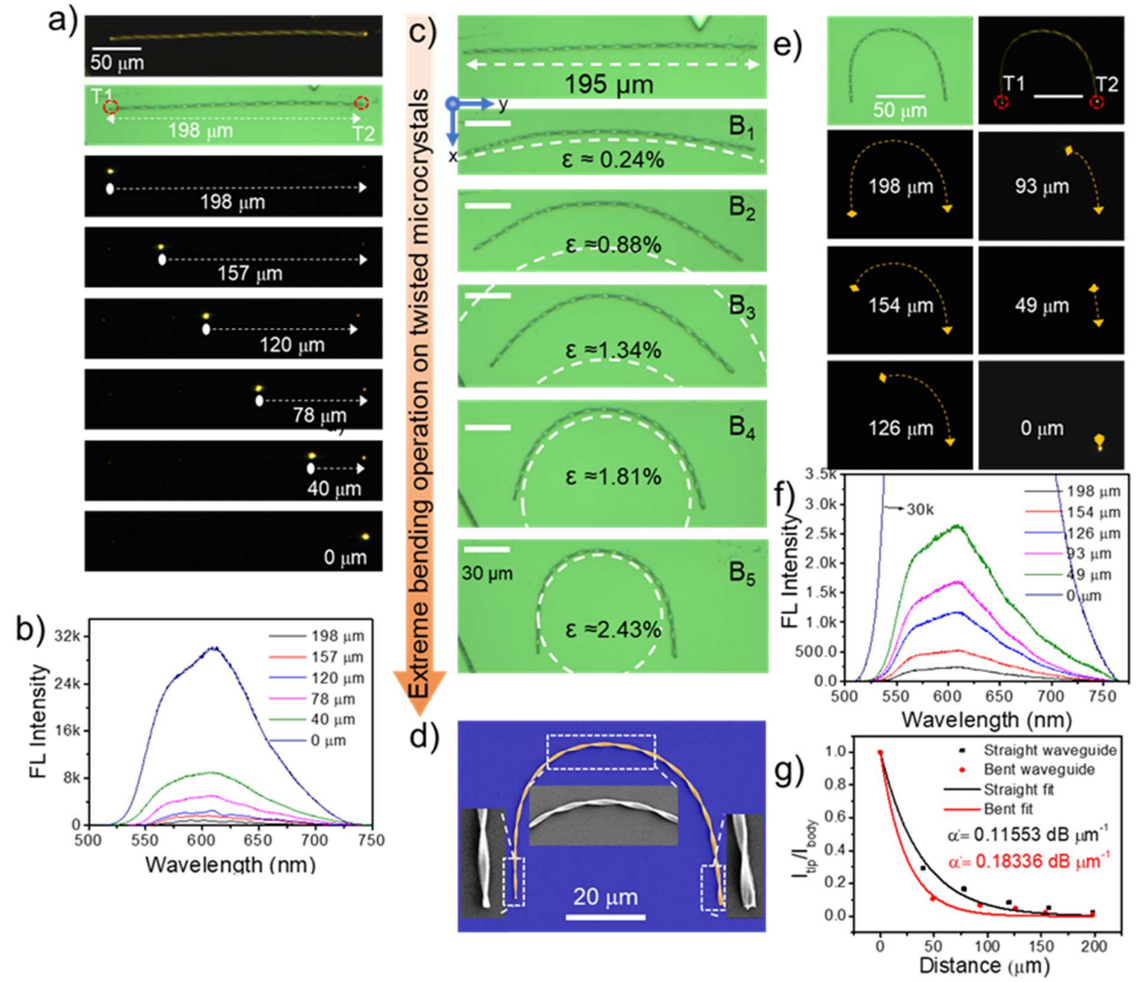

Supplementary Figure 24. Optical waveguiding of straight and bent twisted crystals. Confocal optical and FL images of a) straight and e) bent geometries of twisted crystals (B1-B5) exciting with 405 nm laser at different positions. b,f) FL spectra of corresponding excitation position-dependent waveguiding for a) straight and e) B5 bent geometry. c) Optical images of a straight twisted microcrystal of length ≈195 μm with its subsequent bent geometries (B1-B5) and respective mechanical strain (ε). Scale bar is 30 mm. d) FESEM image of corresponding B4 geometry. g) A plot of the I_{tip}/I_{body} versus the distance of the light propagation path used to estimate the optical loss coefficient (α') for B5 twisted waveguides.

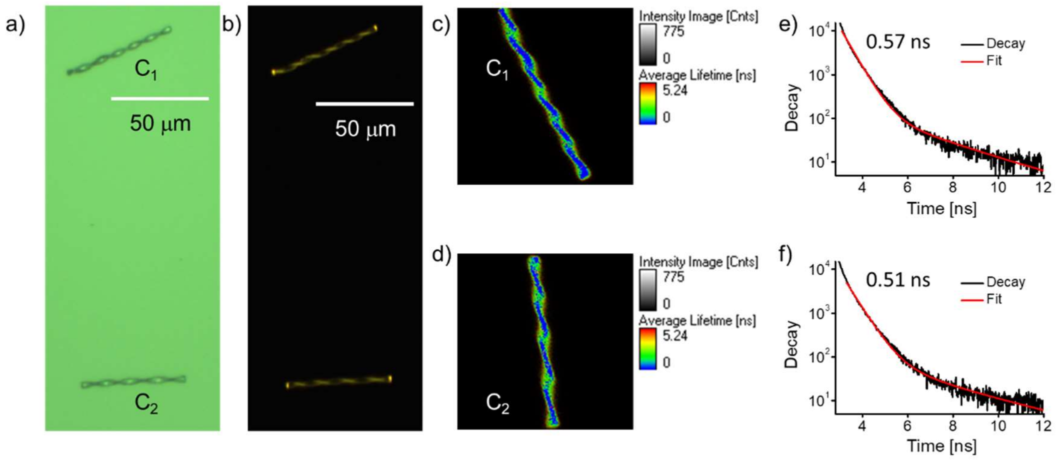

Supplementary Figure 25. FL lifetime image of twisted crystals. a) Confocal and b) its corresponding FL image of twisted microcrystals. c,d) FL lifetime image and e,f) its corresponding decay plots of twisted microcrystals.

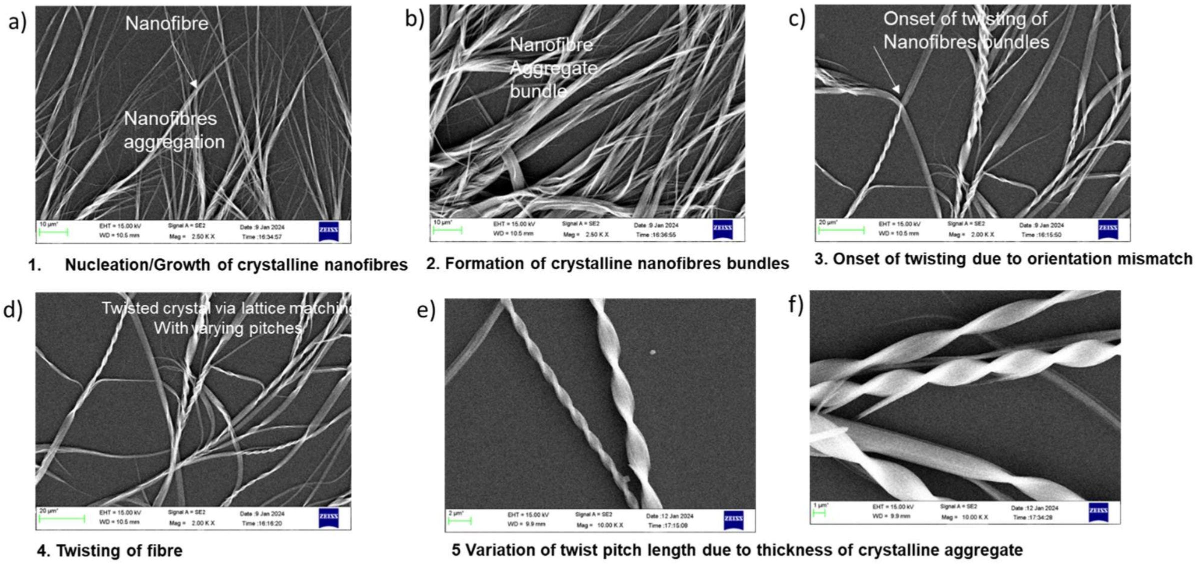

Supplementary Figure 26. Growth mechanism of twisted crystals. a-f) FESEM images recorded at different experiments displaying the growth of twisted crystals. Representative videos on growth of twisted crystals are shown in **Supplementary movies 3 and 9.**
