# Supplementary material for: Mechanically controlled multifaceted dynamic transformations in twisted organic crystal waveguides
Source: Nat Commun. 2024 May 13;15:4040. doi: 10.1038/s41467-024-47924-y (PMC11091112; doi:10.1038/s41467-024-47924-y)
Supplement: Supplementary file 3 — Description of Additional Supplementary Files [file 41467_2024_47924_MOESM3_ESM.pdf]

Description of Additional Supplementary Files

File Name: Supplementary Movie 1
Description: Bending of crystal which is not twisted.

File Name: Supplementary Movie 2
Description: Ending of crystal which is not twisted.

File Name: Supplementary Movie 3
Description: Growth mechanism of twisted crystal.

File Name: Supplementary Movie 4
Description: Microscale flexibility of twisted crystal.

File Name: Supplementary Movie 5
Description: Standing of 8 twisted microcrystals.

File Name: Supplementary Movie 6
Description: Stacking of twisted microcrystals.

File Name: Supplementary Movie 7
Description: Interlocking of twisted microcrystals.

File Name: Supplementary Movie 8
Description: Rolling of interlocked microcrystals.

File Name: Supplementary Movie 9
Description: Growth of twisted crystal.

File Name: Supplementary Movie 10
Description: Cutting and lifting of twisted crystals, and dropping at different substrates, and make them stand.
